# Tetranuclear Polypyridylruthenium(II) Complexes as Selective Nucleic Acid Stains for Flow Cytometric Analysis of Monocytic and Epithelial Lung Carcinoma Large Extracellular Vesicles

**DOI:** 10.3390/biom14060664

**Published:** 2024-06-06

**Authors:** Kartika Wardhani, Aviva Levina, Biyun Sun, Haipei Zou, Georges E. R. Grau, F. Richard Keene, J. Grant Collins, Peter A. Lay

**Affiliations:** 1 School of Chemistry, The University of Sydney, Sydney, NSW 2006, Australia; kwardhani@lanl.gov (K.W.); haipei.zou@outlook.com (H.Z.); 2 Biochemistry and Biotechnology (B-TEK) Group, Bioscience Division, Los Alamos National Laboratory, Los Alamos, NM 87545, USA; 3 School of Natural Sciences, Faculty of Science and Engineering, Macquarie University, Sydney, NSW 2109, Australia; biyun.sun@mq.edu.au; 4 Sydney Nano, The University of Sydney, Sydney, NSW 2006, Australia; georges.grau@sydney.edu.au; 5 Sydney Cancer Network, The University of Sydney, Sydney, NSW 2006, Australia; 6 Marie Bashir Institute, The University of Sydney, Sydney, NSW 2006, Australia; 7 Vascular Immunology Unit, School of Medical Sciences, Faculty of Medicine and Health, The University of Sydney, Sydney, NSW 2006, Australia; 8 Discipline of Chemistry, School of Physics, Chemistry, and Earth Sciences, The University of Adelaide, Adelaide, SA 5005, Australia; 9 Australian Institute of Tropical Health and Medicine/Centre for Molecular Therapeutics, James Cook University, Townsville, QLD 4811, Australia; 10 School of Science, The University of New South Wales, Australian Defence Force Academy, Canberra, ACT 2612, Australia; grant.collins@unsw.edu.au; 11 Sydney Analytical, The University of Sydney, Sydney, NSW 2006, Australia

**Keywords:** ruthenium phosphorescent complexes, selective extracellular vesicle stain, microvesicles, flow cytometry, nucleic acids

## Abstract

Selective staining of extracellular vesicles (EVs) is a major challenge for diagnostic and therapeutic applications. Herein, the EV labeling properties of a new class of tetranuclear polypyridylruthenium(II) complexes, Rubb_7_-TNL and Rubb_7_-TL, as phosphorescent stains are described. These new stains have many advantages over standard stains to detect and characterize EVs, including: high specificity for EV staining versus cell staining; high phosphorescence yields; photostability; and a lack of leaching from EVs until incorporation with target cells. As an example of their utility, large EVs released from control (basal) or lipopolysaccharide (LPS)-stimulated THP-1 monocytic leukemia cells were studied as a model of immune system EVs released during bacterial infection. Key findings from EV staining combined with flow cytometry were as follows: (i) LPS-stimulated THP-1 cells generated significantly larger and more numerous large EVs, as compared with those from unstimulated cells; (ii) EVs retained native EV physical properties after staining; and (iii) the new stains selectively differentiated intact large EVs from artificial liposomes, which are models of cell membrane fragments or other lipid-containing debris, as well as distinguished two distinct subpopulations of monocytic EVs within the same experiment, as a result of biochemical differences between unstimulated and LPS-stimulated monocytes. Comparatively, the staining patterns of A549 epithelial lung carcinoma-derived EVs closely resembled those of THP-1 cell line-derived EVs, which highlighted similarities in their selective staining despite their distinct cellular origins. This is consistent with the hypothesis that these new phosphorescent stains target RNA within the EVs.

## 1. Introduction

Large extracellular vesicles (large EVs, mainly microvesicles, MVs) [1] are crucial for both normal physiological responses and disease progression [2,3,4]. Large EVs serve functionally as mediators of cell-to-cell communication through juxtacrine signaling, or by disseminating various genetic cargos and bioactive molecules of their original parent cells to neighboring and distant sites [4,5,6]. Due to their multiple crucial roles in extracellular signaling, chemical (fluorescent) staining of large EVs is indispensable to monitor their release, extracellular transport, and interactions with target cells [7,8].

Selective staining of intact EVs remains a challenge to understand their biological roles, including physiological and pathological functions, clinical potential, and the intracellular fate of their molecular components [7,9,10]. Current fluorescent EV staining strategies have limitations, including low selectivity and throughput [10,11,12,13]. For instance, prevailing fluorescent and other labeling or staining strategies for isolated EVs utilize lipid membrane-, surface protein-, luminal-, nucleic acid-, radionuclide-, and quantum dot (QD)-labels for visualization and characterization of EVs [10,14]. Staining of isolated EVs with fluorescent lipophilic membrane dyes is very simple to perform but generally lacks selectivity and specificity [10,15]. Surface protein labeling promotes more selective staining of EVs than lipid membrane labeling; however, the conjugation of fluorescent protein labels can similarly influence interactions of EVs with recipient cells [10,16]. For luminal labeling, it is still not established whether all classes of EVs contain esterase activity [10,17]. RNA and DNA labeling strategies offer greater sensitivity to target and stain EVs, but fluorescent probes that intercalate polynucleic acids can affect and disrupt their physical properties and biochemical processes [10,18]. While QDs possess many desirable properties, potential toxic effects and radiation safety concerns related to QDs and radionuclide tracer materials remain significant for EV labeling [10,19]. Consequently, there is a need for new and improved selective fluorescent and phosphorescent stains to overcome the shortcomings of existing EV labeling strategies. These issues include the ability to distinguish intact EVs from cellular debris and other non-EV components [10,20]. Considering the limited application of polynucleic acid-specific stains for EVs, it is desirable to direct further attention to this labelling strategy. EV-associated polynucleic acids (DNA/RNA) play a critical role in a wide range of specific biological and physiological phenomena, which highlights the significance of investigating this approach [10].

**Figure 1:**
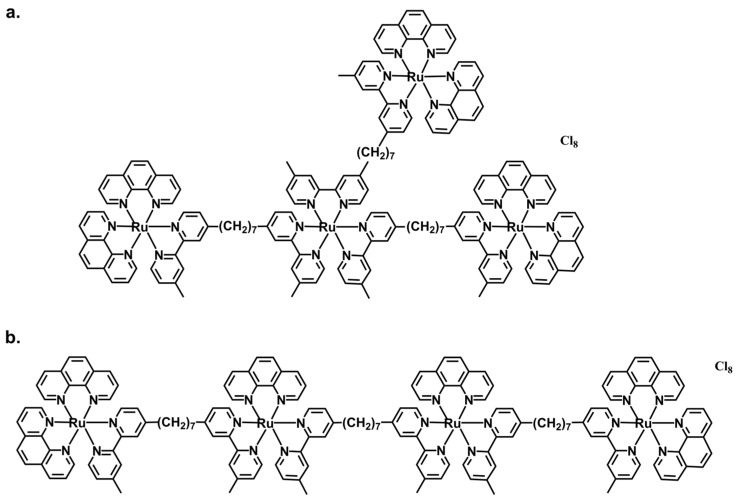
Structures of tetranuclear polypyridylruthenium(II) complexes (**a**) Rubb_7_-TNL and (**b**) Rubb_7_-TL, used for selective nucleic acid staining of intact monocytic (THP-1) and lung cancer cell (A549) EVs.

Polypyridylruthenium(II) complexes are well-recognized stains of DNA and RNA for bioimaging of live cells and have played an important role as anti-cancer agents along with other N-heterocyclic Ru complexes in which binding to human serum albumin plays a role [21,22,23,24]. While tetranuclear polypyridylruthenium(II) complexes have a high binding affinity to human serum albumin, they can enter cells where they localize in the nucleolus of living cells [25,26,27], which is a region of high RNA concentration, and in the cytoplasm, but do not co-localize in the mitochondria [25,26,27]. This indicated a potential for EV staining applications because of their relatively high RNA cargoes, particularly mRNA, which has a greatly enhanced concentration in EVs versus the parent cells from which they are derived [28,29,30]. Previously, a new non-linear tetranuclear polypyridylruthenium(II) complex, Rubb_7_-TNL, was shown to be an effective stain for monitoring the effects of EVs on a model of the blood-brain barrier (BBB) [20,31]. In the current study, the aim is to investigate comprehensively the staining properties of Rubb_7_-TNL and its linear analogue, Rubb_7_-TL. Both complexes contain flexible bis [4(4′-methyl-2,2′-bipyridyl)]-1,7-heptane bridges but different arrangements of the Ru moieties (Figure 1) [20,25,26,27,31], which enabled us to determine whether linear or branched complexes were better stains. In particular, we aimed to elucidate the sensitivity and selectivity of these new complexes in EV staining applications, which could offer new techniques for studying EV biology and their roles in various physiological and pathological conditions. As an example of the application of these new stains, flow cytometry studies of monocytic large EVs released from control versus lipopolysaccharide (LPS)-stimulated THP-1 monocytes were compared to a model immune system EV response to bacterial infection [20,31,32]. Furthermore, both Rubb_7_-TNL and Rubb_7_-TL were utilized to stain EVs isolated from A549 epithelial lung carcinoma cells and were compared with the staining of monocytic EVs to validate the selectivity of these new complexes as polynucleic acid stains for EVs originating from very different cell types.

## 2. Materials and Methods

### 2.1. Materials and Stock Solutions

Tetranuclear polypyridylruthenium(II) complexes-based stains, Rubb_7_-TNL and Rubb_7_-TL (Figure 1) were synthesized and characterized, as previously described [25,26,27]. Stock solutions of Ru complexes (10 mM Ru) in high-purity (>99.9%) dimethyl sulfoxide (DMSO, Merck Millipore, Darmstadt, Germany, Cat. No. 5439001000) were stored at 295 K, protected from light and moisture; their stability over time (at least six months) was verified by electronic absorption spectroscopy [25,26,27]. Other Analytical-grade (>99% purity) reagents and HPLC grade solvents from Sigma-Aldrich, Saint Louis, MO, USA or Merck Millipore, Darmstadt, Germany were used without further purification, and water was purified by the Milli-Q technique.

### 2.2. Cell Culture

The human monocyte cancer cell line THP-1 (human acute monocytic leukemia, ATCC, Manassas, VA, USA, Cat. No. TIB-202) was purchased from the American Type Culture Collection (ATCC, Manassas, VA, USA). Pre-sterilized solutions and plasticware for mammalian cell culture were purchased from Thermo Fisher Scientific (TFS, Cambridge, MA, USA), unless specified otherwise. The cell line was cultured in a sterile environment using standard techniques [33,34,35] in advanced RPMI 1640 (TFS, Cambridge, MA, USA, Cat. No. 12633012), supplemented with GlutaMax (TFS, Cambridge, MA, USA, Cat. No. 35050061; equivalent to 2.0 mM L-glutamine), an antibiotic-antimycotic mixture (TFS, Cambridge, MA, USA, Cat. No. 15240062; 1.0 U mL^−1^ penicillin, 1.0 mg mL^−1^ streptomycin, and 2.5 µg mL^−1^ amphotericin B), and fetal calf serum (FCS; Gibco, New York, NY, USA, Cat. No. 10500064; 2.0% vol.), which is referred to as the growth medium. Cell density and viability were assessed regularly by the trypan blue assay (TFS, Cambridge, MA, USA, Cat. No. T10282) together with the Invitrogen Countess Automated Cell Counter (TFS, Cambridge, MA, USA).

The human lung cancer line A549 (epithelial lung carcinoma, ATCC, Manassas, VA, USA, Cat. No. CCL-185™) cells were grown to confluence in 75 cm^2^ cell culture plates in Advanced Dulbecco’s modified Eagle medium (Advanced DMEM; TFS, Cambridge, MA, USA, Cat. No. 12491-015) + 2% FCS, then the medium was replaced with serum-free medium (Advanced DMEM + 1.0 mg mL^−1^ AlbuMax, lipid-rich bovine serum albumin, TFS, Cambridge, MA, USA, Cat. No. 11020021).

### 2.3. Isolation of Monocytic EVs from Immune System THP-1 Cells and A549-EVs from Human Lung Cancer A549 Cells

Typical THP-1 monocytes were grown to a density of 2 × 10^6^ cells mL^−1^ in growth medium (20 mL) within 175 cm^2^ cell culture flasks. The cells were pelleted by centrifugation (2 min at 600× *g*) and re-suspended in 20 mL of serum-free medium (same as the growth medium, but FCS was entirely replaced with 1.0 mg mL^−1^ of AlbuMax, lipid-rich bovine serum albumin, TFS, Cambridge, MA, USA, Cat. No. 11020021). Serum-free medium was used to avoid the interference of EVs that are present in serum. The resultant suspension was divided between two 9-cm cell culture dishes (10 mL per dish), and bacterial lipopolysaccharide (LPS, *Escherichia coli*; Sigma-Aldrich, Saint Louis, MO, USA, Cat. No. L6529; concentration in the medium 100 ng mL^−1^) was added into one of the dishes, while the second one was left as a control (basal). The dose of LPS (100 ng mL^−1^) was selected as the optimal dose for monocytic EV release without significantly compromising THP-1 parental cell viability [32,36]. After overnight incubation, stimulated and control cell suspensions were collected into separate centrifuge tubes, and cells were removed by centrifugation (2 min at 600× *g*). The supernatants were then sequentially centrifuged for 5 min at 1200× *g* (295 K) to remove the cell debris and for 60 min at 20,000× *g* (277 K) to pellet the monocytic EVs, as previously described [32,36]. The monocytic EV pellets were re-suspended in 5.0 mL of phosphate-buffered saline (PBS, Ca^2+^ and Mg^2+^ free; TFS, Cambridge, MA, USA, Cat. No. 10010-023) and re-centrifuged (60 min at 20,000× *g* and 277 K) [32,36]. The resultant monocytic EV pellets were re-suspended in 0.20 mL of PBS and kept frozen at 253 K until they were prepared for staining and further characterization [20,32,36].

After overnight incubation of the A549 cells, the serum-free medium was collected and centrifuged for 5 min at 1200× *g* (295 K) to remove the cell debris. The supernatant was then sequentially centrifuged for 60 min at 20,000× *g* (277 K) to pellet the A549-EVs. The A549-EV pellets were re-suspended in 5.0 mL of PBS and re-centrifuged (60 min at 20,000× *g* and 277 K) [32,36]. The resultant A549-EV pellets were re-suspended in 0.20 mL PBS and kept frozen at 253 K until they were prepared for staining and further characterization [20,32,36].

### 2.4. Dynamic Light Scattering (DLS) Measurements

**Figure 2:**
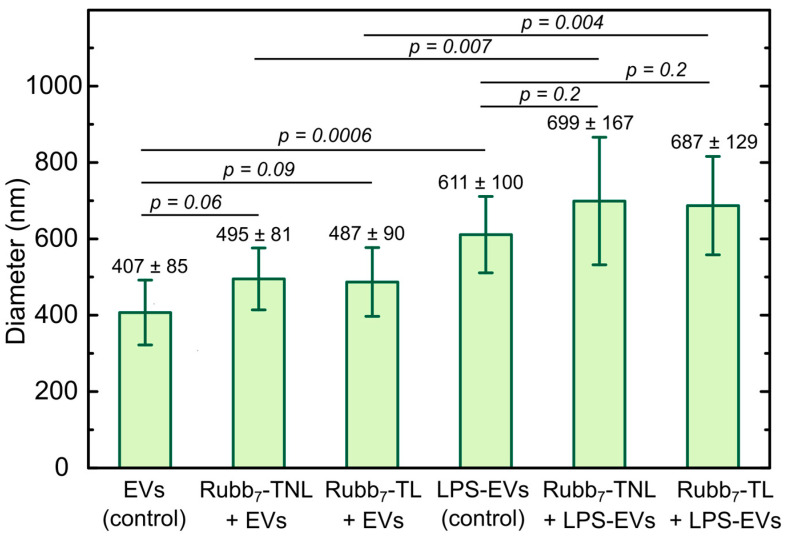
DLS particle size distributions of monocytic large EVs released by unstimulated and LPS-stimulated THP-1 monocytic leukemia cells before and after staining (1 h with 10 µM of Rubb_7_-TNL or Rubb_7_-TL at 298 K, room temperature). Results are expressed as the mean ± standard deviation of eight independent experiments for each treatment group. EV samples were isolated from eight different batches (*n* = 8) of unstimulated and LPS-stimulated THP-1 cells. The statistical significance of differences between two sets of data was measured using a two-sample independent *t*-test (calculated using Origin 6.1 software). The minimal requirement for statistical significance was set at *p* < 0.05.

The particle size distribution of the monocytic EV pellets suspended in 0.20 mL PBS, released by unstimulated and LPS-stimulated THP-1 cells, before and after staining with polypyridylruthenium(II) phosphophores, was measured by DLS using the Malvern ZetaSizer NanoS instrument (173° scattering angle, 298 K, Malvern, UK) and Malvern ZEN0040 disposable cuvettes (Figure 2). The measured parameters were the averages of 12–15 scans (scan time, 3 s). For the A549-EVs, size distribution measurements by DLS were performed before and after staining using the same procedures and parameters as aforementioned for monocytic EV suspensions.

### 2.5. Staining of Isolated Monocytic EVs and A549-EVs with Polypyridylruthenium(II) Phosphophores

**Figure 3:**
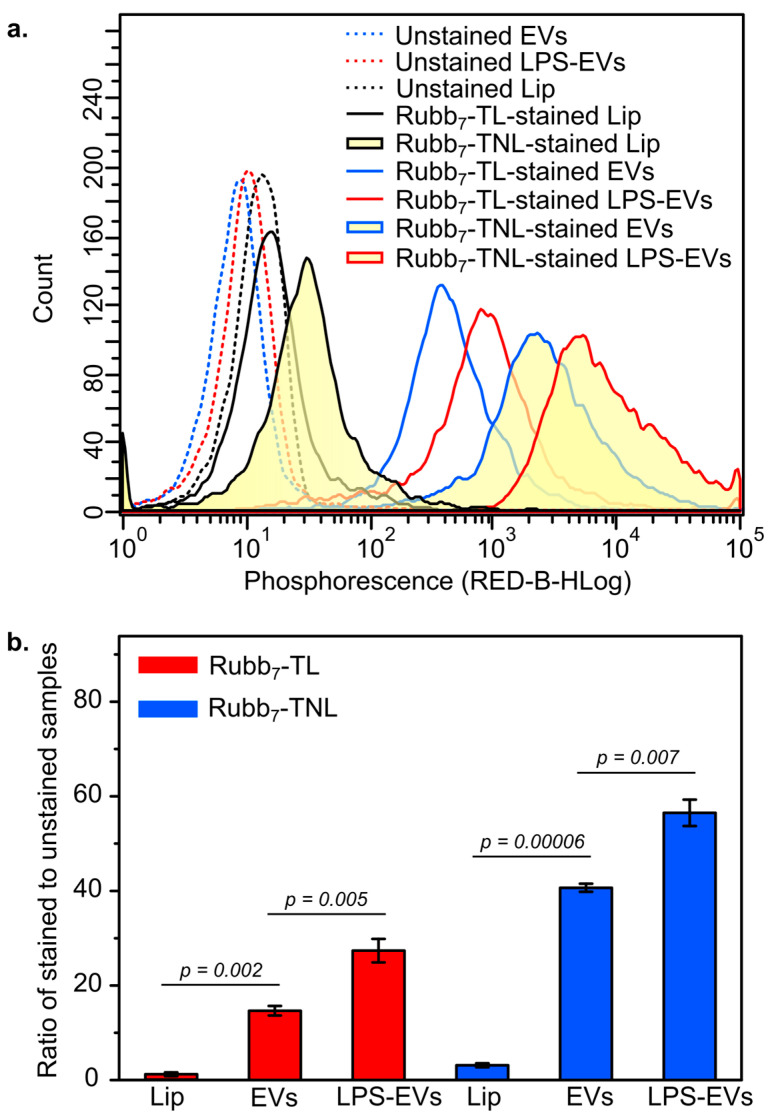
Comparison of phosphorescence for representative samples of unstimulated and LPS-stimulated EVs isolated from THP-1 monocytic leukemia cells and artificial DOPC/cholesterol liposomes in PBS. (**a**) Samples stained with 10 µM of Rubb_7_-TNL or Rubb_7_-TL in PBS for 1 h at 298 K (room temperature), followed by removal of excess Ru-stain. (**b**) Ratios of median phosphorescence of stained and unstained vesicles. Error bars show the standard deviation of the ratios of five independent experiments for each treatment group. EV samples were isolated from five different batches (*n* = 5) of unstimulated and LPS-stimulated THP-1 cells. The statistical significance of differences between two sets of data was measured using a two-sample independent *t*-test (calculated using Origin 6.1 software). The minimal requirement for statistical significance was set at *p* < 0.05.

Optimization of staining conditions for monocytic EVs released from unstimulated and lipopolysaccharide (LPS)-stimulated THP-1 cells was performed. Both unstimulated and LPS-stimulated EVs were stained with either Rubb_7_-TNL or Rubb_7_-TL at final ruthenium concentrations of 1, 2, 5, and 10 µM in phosphate-buffered saline (PBS) for 0.5 h at 295 K, followed by removal of excess stain. Notable changes in phosphorescence intensity were observed with a final Ru concentration of 10 µM at different staining times (0.5 and 1.0 h). As outlined previously, optimal staining conditions were determined to be 1 h of staining with 10 µM of Rubb_7_-TNL or Rubb_7_-TL [20], which resulted in visible separation of phosphorescence intensity between unstimulated and LPS-stimulated EV subpopulations (Figure 3). As a result, all experiments reported in this manuscript used these conditions, as detailed below.

Typically, 1.0 µL of Rubb_7_-TNL or Rubb_7_-TL solutions in DMSO (2.0 mM Ru) was added to 0.20 mL of monocytic EVs suspension in PBS (final Ru concentration, 10 µM), and the suspensions were kept in the dark for 1 h at 298 K (room temperature). After that, stained monocytic EVs were pelleted by centrifugation (60 min at 20,000× *g* and 277 K) and washed twice with 1.0 mL PBS by re-suspending and re-centrifugation under the same conditions. The purified, stained monocytic EV pellets were re-suspended in 0.20 mL of PBS and stored at 253 K until they were used for flow cytometric analysis (Figure 3). Ru uptake by EVs was measured in three random samples of EVs released from LPS-stimulated THP-1 cells. Each EV pellet was lysed in 0.10 mL of 0.10 M NaOH (overnight at 277 K), and an aliquot of the lysate (5.0 μL) was used for protein content determination using the Bradford assay with bovine serum albumin (BSA) as a standard. The remaining lysate was mixed with 0.10 M HCl (0.90 mL), and the Ru content in the resultant solution was determined by ICPMS (Perkin-Elmer Nexion 350X spectrometer, PerkinElmer, Inc., Waltham, MA, USA) using a standard Ru solution (Sigma-Aldrich, Saint Louis, MO, USA, Cat. No. 207446) and ^193^Ir peak as an internal standard. The measured values of Ru uptake by EV during staining with Rubb_7_-TNL were 12 ± 4 nmol Ru per mg protein (average and standard deviation for three separate EV samples).

**Figure 4:**
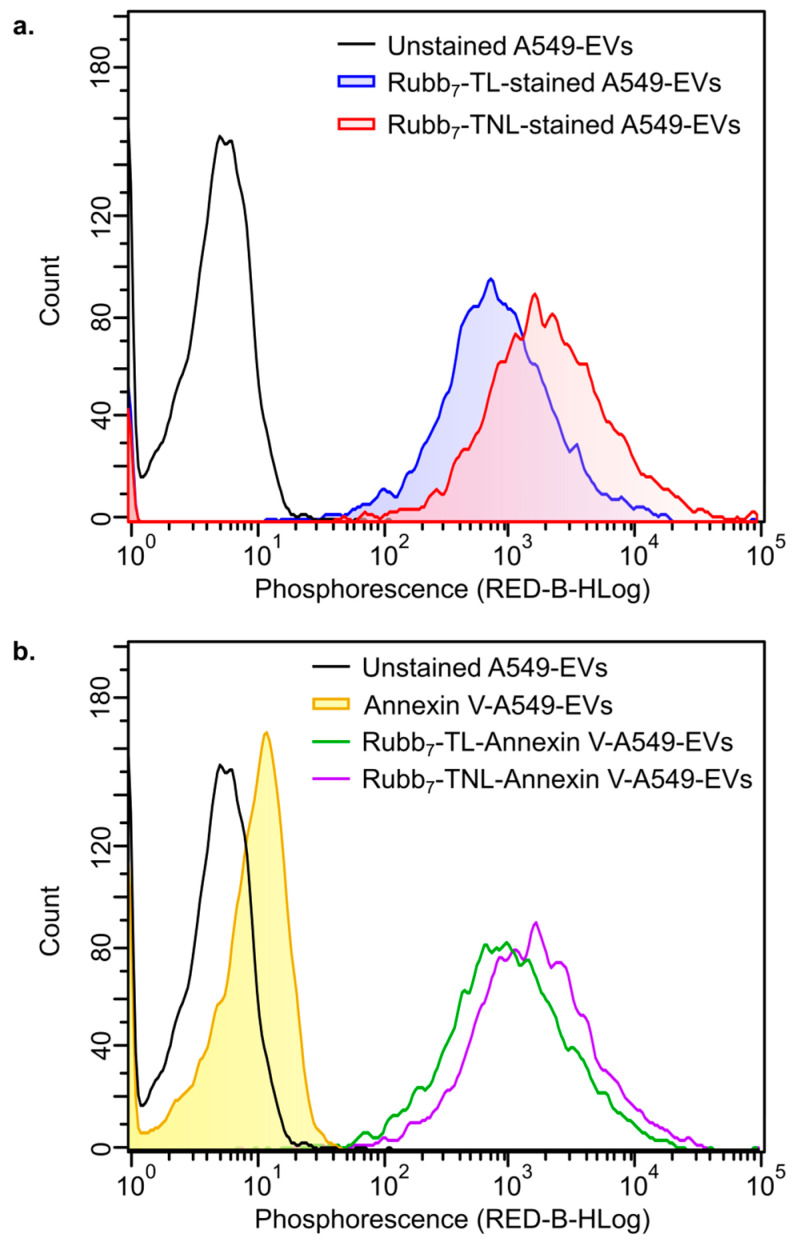
Comparison of phosphorescence for representative samples of EVs isolated from A549 epithelial lung carcinoma cells: (**a**) A549-EV samples were unstained and stained with 10 µM of Rubb_7_-TNL or Rubb_7_-TL in PBS for 1 h at 298 K (room temperature), followed by removal of excess Ru-stain, without Annexin V-FITC treatment, and (**b**) samples were additionally treated with Annexin V-FITC solution in binding buffer for 30 min at 298 K.

Phosphorescent staining of A549-EVs with Rubb_7_-TNL or Rubb_7_-TL were performed using the same procedures for monocytic EV suspensions. For representative comparison, two samples of A549-EVs were prepared for each condition: (i) unstained A549-EVs control; (ii) Rubb_7_-TNL-stained A549-EVs; and (iii) Rubb_7_-TL-stained A549-EVs. One sample from each condition was directly used for flow cytometry, while the second sample from both the unstained and Ru-stained solutions was stained with Annexin V immediately prior to flow cytometry (Beckman Coulter IM3614 kit, Beckman Coulter, Brea, CA, USA). For Annexin V staining, 0.20 mL of A549-EV suspensions were mixed with 20 µL of 10X binding buffer and 10 µL of Annexin V-FITC solution, and the resulting solution was kept for 30 min at 298 K before flow cytometry (Figure 4).

### 2.6. Preparation and Staining of Artificial Liposomes

Multi-lamellar DOPC-cholesterol 1:1 liposomes (where DOPC is dioleoylphosphatidylcholine), which are widely used as models of mammalian cell membranes [37], were prepared according to a well-established procedure [38]. Concentrated liposome suspensions (~3 mM lipid in 50 mM Tris buffer, 150 mM NaCl, pH 7.0) were stored at 277 K and vigorously mixed by vortexing and diluted 200-fold with PBS for further analyses. Size distribution measurements by DLS and staining with Ru stains were performed using the same procedures aforementioned for monocytic EV suspensions.

### 2.7. Analysis of Stained Monocytic EVs, A549-EVs, and Artificial Liposomes by Flow Cytometry

Analyses were performed using a Guava EasyCyte 6–2 L benchtop flow cytometer (Merck Millipore, Darmstadt, Germany). Typical flow cytometer settings for monocytic EVs (Figure 3) were as follows: 488 nm laser on; green, yellow, and red emission channels on (were set to high detection level, gain 10); forward (FCS) and side (SSC) scattering gain, 10; FCS threshold, 0; flow rate, 0.12 μL s^−1^; maximal counting of 5000 gated events. Suspensions of stained or unstained EV or liposomes in 0.20 mL of PBS were used for flow cytometry (Figure 3). Flow cytometer settings for A549-EVs (Figure 4) were as follows: 448 nm and 642 nm lasers on; green (GRN-B, gain 14.3), yellow (YEL-B, gain 11.8), and red (RED-B, gain 13.5; RED-R, gain 30.6) emission channels on, were set to high detection levels; FCS and SSC scattering gain, 11.3 and 11.3; FCS threshold, 100; flow rate, low; maximal counting 5000 gated events. Blank PBS solution was used to gate out the electronic noise, and 0.46 μm and 1.1 μm polystyrene beads (Sigma-Aldrich, Saint Louis, MO, USA, Cat. No. LB5 and LB11) were used for calibration, as described previously [39]. All the plots were gated to exclude non-specific background “electronic noise” (measured using PBS as the background).

For all experiments described in this paper, consistent results were reproduced in multiple independent experiments. The same procedures and instrument settings were applied to avoid interexperimental variations.

## 3. Results

### 3.1. Effects of Polypyridylruthenium(II) Phosphophores on the Size of Extracellular Vesicles

To confirm that the size of biological particles used in this study was consistent with that of EVs, freshly isolated monocytic EVs under unstimulated (basal) and LPS-stimulated conditions were subjected to dynamic light scattering (DLS) measurements. The particle size distributions of all monocytic EV samples were within the accepted size range for cellular EVs (Figure 2) [2,40]. Consistent with previous studies [32,36,41,42], THP-1 monocytes stimulated by bacterial LPS produced significantly larger monocytic EVs (611 ± 100 nm, *p* = 0.0006) compared with those from unstimulated cells (407 ± 85 nm). This increase in size reflects a change in the biomolecular content of monocytic large EVs, as previously described [32,36,41,42].

The effects of Rubb_7_-TNL and Rubb_7_-TL on the size of monocytic EVs, before and after staining, were also assessed by DLS (Figure 2). For both stains, particle size distributions showed that EVs from both unstimulated and LPS-stimulated THP-1 were still EV-sized without significant increase in vesicle size compared to unstained, which were 495 ± 81 nm (*p* = 0.06), 699 ± 167 nm (*p* = 0.2), 487 ± 90 nm (*p* = 0.09), and 687 ± 129 nm (*p* = 0.2) for Rubb_7_-TNL-stained EVs, Rubb_7_-TNL-stained LPS-stimulated EVs, Rubb_7_-TL-stained EVs, and Rubb_7_-TL-stained LPS-stimulated EVs, respectively.

For EVs isolated from the A549 lung cancer line, the average EV size was as follows: 403 ± 163 nm, 487 ± 137 nm, 376 ± 87 nm, 379 ± 146 nm, 507 ± 109 nm, and 491 ± 92 nm (all data *p* < 0.05) for unstained A549-EVs, Rubb_7_-TL-stained A549-EVs, Rubb_7_-TNL-stained A549-EVs, Annexin V-stained A549-EVs, Rubb_7_-TL-Annexin V-stained A549-EVs, and Rubb_7_-TNL-Annexin V-stained A549-EVs, respectively. The DLS measurements showed no obvious effects of Ru-dye staining, and these sizes were comparable to those of monocytic EVs released from unstimulated THP-1 cells [32,36,41,42].

### 3.2. Analysis of Polypyridylruthenium(II) Phosphophores-Stained Monocytic Extracellular Vesicles and Liposomes by Flow Cytometry

Flow cytometry is complementary to DLS and provides additional information on the numbers of EVs, density, and phosphorescence intensities of individual and sub-population distributions of EVs [8,39]. The calibration using PBS and latex nanospheres (beads) showed that conventional flow cytometry detected individual EVs (Figure S1) [39,43]. Flow cytometric analysis demonstrated that EVs from LPS-stimulated THP-1 cells had higher granularity on both the FSC and SSC plots due to increased size and complexity, respectively, as compared to the subpopulation of large EVs from unstimulated monocytes (Figure S2). These results were consistent with the size increase measured by DLS (Figure 2). Optimal staining conditions were 1 h of staining with 10 µM of Rubb_7_-TNL or Rubb_7_-TL (final Ru concentration), which resulted in visible separation of phosphorescence intensity between unstimulated and LPS-stimulated EV subpopulations (Figure S3).

Flow cytometric analysis showed that both Rubb_7_-TNL and Rubb_7_-TL are excellent stains for EVs, as shown by much higher phosphorescence intensities in comparison with unstained controls (Figure 3a). Rubb_7_-TNL had higher phosphorescence intensities than did Rubb_7_-TL for all EV samples (Figure 3a and Figure S4). This trend resulted in a higher ratio of the median phosphorescence values of stained versus unstained samples (Figure 3b). The more lipophilic Rubb_7_-TNL concentrated more efficiently and/or was more phosphorescent when bound to RNA within EVs than was Rubb_7_-TL (lipophilicity, log*P* values as chloride salts: −1.6 and −1.9, respectively) [25]. Lipophilicity is a key determinant of the accumulation of a Ru complex within EVs to enable the EV to penetrate the membrane efficiently and interact with biochemical target(s) [25].

To further validate the selectivity of these novel phosphophores in intact EVs, DOPC/cholesterol liposomes that model EV membranes [44] but lack all of the other components of EVs, e.g., nucleic acids and proteins (“empty EVs”), were performed using the same procedures for staining EVs. DLS showed the average size of the artificial liposomes (400 ± 80 nm, *n* = 3) was comparable with that of EVs. As expected, in the presence of the Ru phosphophores, there was very little phosphorescence intensity (no selectivity) from liposomes compared to high phosphorescence from monocytic large EVs treated under the same conditions (Figure 3a,b and Figure S4). These results confirmed that both Rubb_7_-TNL and Rubb_7_-TL had high selectivity for intact monocytic EVs compared with cell membrane fragments or debris. Moreover, Rubb_7_-TL exhibited lower peak phosphorescence staining of DOPC/cholesterol liposomes than did Rubb_7_-TNL (Figure 3a,b), which showed that Rubb_7_-TL was slightly more selective in staining of intact monocytic large EVs compared to liposomes and cell debris. It is postulated that the lower selectivity of Rubb_7_-TNL for EVs was due to a slightly higher tendency to stain lipids, as Rubb_7_-TNL is slightly more lipophilic than Rubb_7_-TL [25].

### 3.3. Analysis of Polypyridylruthenium(II) Phosphophores-Stained A549 Lung Cancer Extracellular Vesicles by Flow Cytometry

In order to determine the ability of the stains to label EVs from other cell lines, preliminary flow cytometric analyses were undertaken with EVs isolated from A549 lung cancer cells, using the same isolation protocol. Optimal staining conditions have yet to be conducted with these A549-EVs, but even after 1 h of staining with 10 µM of Rubb_7_-TNL or Rubb_7_-TL (final Ru concentration) at 298 K, which was used for staining monocytic EVs, strong staining was evident (Figure 4a). In addition to this, the Rubb_7_-TNL and Rubb_7_-TL dye staining was compared with Annexin V staining, since the latter is typically used to stain phosphatidylserine (PS) on exposed EV membranes and apoptotic cells [11,13,45]. As expected, in the phosphorescence channel, the use of the Annexin V stain alone (Figure 4b) on A549-EVs resulted in a small shift in peak phosphorescence intensity (lacking selectivity) compared to unstained A549-EVs, but when co-stained with Rubb_7_-TNL or Rubb_7_-TL, there was a large increase in phosphorescence intensity similar to that observed in the absence of the Annexin-V stain (Figure 4a), which is consistent with the two stains targeting different components of the EVs (Figure 3a) [11,13,45]. The Rubb_7_-TNL staining under these conditions gave a more intense high phosphorescence peak (~10^5^) than the Rubb_7_-TL staining (Figure 4a), which is similar to that observed with the monocytic EVs (Figure 3). Also, the Rubb_7_-TNL staining (Figure 4a) appeared to provide better resolution of overlapping peaks, presumably due to different sub-populations of EVs [11,13,45].

## 4. Discussion

In this study, novel polypyridylruthenium(II) phosphophores, Rubb_7_-TNL and Rubb_7_-TL (Figure 1), that remained very stable in biological media [20], were used as selective nucleic acid stains for detection and characterization of intact EVs. As an example of their utility, EVs released from control (basal) or lipopolysaccharide (LPS)-stimulated THP-1 monocytic leukemia cells were studied as a model of immune system response to bacterial infection.

The novel phosphorescent staining combined with DLS measurements and flow cytometry revealed three key findings (Figure 2 and Figure 3). First, LPS-stimulated THP-1 cells generated significantly larger and more numerous monocytic EVs as compared with those from unstimulated cells, consistent with previous studies [32,36,41,42]. Second, both Rubb_7_-TNL and Rubb_7_-TL allowed selective staining and discrimination of two distinct subpopulations of monocytic EVs within the same experiment as a result of biochemical differences derived from unstimulated (basal) and LPS-stimulated THP-1 monocytes. Previous studies showed that EVs released from monocytes under LPS stimulation had significantly increased nucleic acids (mostly as RNA) and proteins and more phosphatidylserine (PS) lipids in their membrane, as compared with those released under unstimulated conditions [32,36]. Hence, the higher phosphorescence intensity from LPS-stimulated EVs was consistent with RNA being the binding site for Rubb_7_-TNL and Rubb_7_-TL (Figure 3a,b and Figure S4). Additionally, DLS analysis confirmed the size distribution of monocytic EVs did not significantly differ between before and after staining with Rubb_7_-TNL or Rubb_7_-TL. This size information is a key consideration as it affects the way EVs mediate intercellular communication [6,9,46,47,48,49]. Previous studies have shown that the use of PKH dye molecules for EV labeling resulted in larger EVs after labeling, which was caused by the aggregation of PKH dyes with EVs as characterized by DLS, nanoparticle tracking analysis (NTA), and flow cytometry [47]. The significant size change of EVs after labeling with PKH changed the cellular uptake level, internalization mechanism, and the biodistribution of EVs [47]. These factors combined reduce the validity of the use of the PKH dye as a lipophilic fluorescent dye for EVs [47], compared with the studies with the Ru dyes. The current study demonstrated that both Rubb_7_-TNL and Rubb_7_-TL are reliable and selective stains for monocytic EVs, while still retaining the native physical properties of monocytic EVs after staining (e.g., size and population) and also preserving phosphorescent detection by flow cytometry [20,31]. The stained EVs did not affect the uptake of the monocytic large EVs by a number of different cell lines, and this will be described in upcoming papers, which will specifically detail the phosphorescent EV staining with Rubb_7_-TNL combined with the IncuCyte live imaging system, used to visualize in real-time the release and uptake of EVs originating from immune system THP-1 cells with live MDA-MB-231 breast cancer cells [20]. Finally, the selective nucleic acid staining by both Rubb_7_-TNL and Rubb_7_-TL enabled highly selective discrimination between intact EVs and cell membrane fragments or other lipid-containing debris for the first time, unlike non-selective lipophilic membrane stains [11,13,45], and fluorescent proteins (or antibodies) conjugated to EVs [12,50,51,52]. This specificity for staining EVs was highlighted by the lack of staining of DOPC/cholesterol liposomes that mimic EV membranes [44], but do not contain all of the other components of EVs, such as RNA and proteins (“empty EVs”). This was a major advantage for EV visualization and for performing functional assays with EVs, compared to staining of the EV membrane [11,13,45] or biomolecules within them [12,50,51,52]. Additionally, this new protocol that incorporates the use of polypyridylruthenium(II) complexes for phosphorescent EV staining when combined with flow cytometry has enabled studies on the interactions of EVs with endothelial cells in an in vitro model of the BBB [31].

While less extensive experiments were undertaken with the A549 epithelial lung carcinoma-derived EVs, the results shown that these A549-EVs (Figure 4) are also stained to a similar extent as those from the THP-1 monocytic leukemia cell line (Figure 3), which is a very different cell type. Moreover, Rubb_7_-TNL and Rubb_7_-TL dyes exhibited considerably greater selectivity than annexin V stains for detecting EVs [11,13,45]. This finding is consistent with the hypothesis that both Rubb_7_-TNL and Rubb_7_-TL dyes are selectively targeting the RNA within the EVs [11,13,45].

It is unclear why the non-linear stain, Rubb_7_-TNL, is a stronger stain than its linear isomer, Rubb_7_-TL (Figure 3), but we postulate that this is due to geometric factors that affect both the minor differences in their lipophilicities (Section 3.2) [25] and intermolecular interactions with targets (e.g., RNA). Future studies will focus on further elucidating these mechanisms to better understand the EV staining behavior of these complexes.

## 5. Conclusions and Future Perspectives

In conclusion, Rubb_7_-TNL and Rubb_7_-TL are reliable, selective, and stable stains for intact monocytic large EVs with no significant staining of artificial liposomes or cell membrane debris. They enable the differentiation of two monocytic large EV subpopulations derived from unstimulated and LPS-stimulated immune system THP-1 monocytic leukemia cells. Moreover, staining with Rubb_7_-TNL and Rubb_7_-TL reveals that EVs isolated from A549 epithelial lung carcinoma cells exhibit staining patterns closely aligned with those of monocytic EVs, which confirms the selectivity of both Rubb_7_-TNL and Rubb_7_-TL as polynucleic acid stains for EVs released from diverse cellular origins. These novel phosphorescent stains offer a simple, quick, reproducible, and precise flow-cytometry-based method of direct staining of intact EVs, which has outstanding potential for studies of the interactions and communication between EVs and target cells to be reported elsewhere. This concept opens up unprecedented opportunities to conduct experiments that explore various metal complexes [53] as selective stains of intact EVs from numerous cell types.

Future research could focus on understanding the EV staining mechanism using Rubb_7_-TNL and Rubb_7_-TL stains by investigating how these stains bind with RNA inside EVs. Advanced spectroscopic techniques like X-ray Fluorescence Microscopy (XFM) with a nanoprobe could be used to map any colocalization of the distribution of Ru in Rubb_7_-TNL or Rubb_7_-TL with phosphorus (P) from RNA EVs. Additionally, molecular modeling could be used to simulate the binding interactions between Rubb_7_-TNL or Rubb_7_-TL and cellular RNA within EVs to provide insights into the molecular dynamics of the EV staining process. Titration experiments that systematically vary RNA concentration in EV samples could be used to establish a quantitative correlation between the Rubb_7_-TNL or Rubb_7_-TL signal and RNA abundance.

Considering the diverse lipid composition of EVs [10], the inclusion of phosphatidylethanolamine, phosphatidylserine, and other lipids as negative controls could provide a more comprehensive evaluation of staining selectivity and specificity. However, such additional controls are not required because these lipids are also present in cellular membranes and are not stained when cells are treated with the complexes [25,26,27]. Rather, regions of high RNA concentrations are stained [25,26,27].

We have also described the generation of stained EVs that are released from stained cells [20]. Since the medium in which the cells are grown contains serum, this shows that serum does not interfere with the staining of the EVs. Therefore, stained tumors or spheroids could be implanted in animals and the release of EVs from cells in such tumors could be monitored as the tumor grows or is treated in vivo. To advance other in vivo EV staining, innovative methodologies to overcome challenges, such as Rubb_7_-TNL or Rubb_7_-TL delivery, tissue penetration, and real-time imaging, will be required. This will require delving into the development of targeted stains with enhanced tissue permeability and specificity for EVs. In all of these experiments, the relatively long wavelengths of the phosphorescence emission of these stains will facilitate in vivo emission microscopy and could facilitate real-time visualization of EV dynamics in living organisms. By advancing our comprehension of in vivo EV staining, we anticipate attaining valuable insights into the complex interplay between EVs and their biological environments.

## Data Availability

Original data is available from the authors on request.

## Supplementary Materials

The following supporting information can be downloaded at: https://www.mdpi.com/article/10.3390/biom14060664/s1, Figure S1: Flow cytometry gating strategy for the identification of intact large EVs (mainly microvesicles, MVs); Figure S2: Flow cytometry forward vs. side scatter intensity plots of unstimulated (basal) and LPS-stimulated EVs; Figure S3: Optimization of staining conditions for monocytic EVs released from unstimulated and LPS-stimulated THP-1 cells; Figure S4: Selectivity of Rubb_7_-TNL and Rubb_7_-TL for the detection of intact monocytic EV-related nucleic acids.
