# Supplementary material for: Tetranuclear Polypyridylruthenium(II) Complexes as Selective Nucleic Acid Stains for Flow Cytometric Analysis of Monocytic and Epithelial Lung Carcinoma Large Extracellular Vesicles
Source: Biomolecules. 2024 Jun 6;14(6):664. doi: 10.3390/biom14060664 (PMC11202172; doi:10.3390/biom14060664)
Supplement: Supplementary file 1 [file biomolecules-14-00664-s001.zip › biomolecules-2752938-supplementary.pdf]

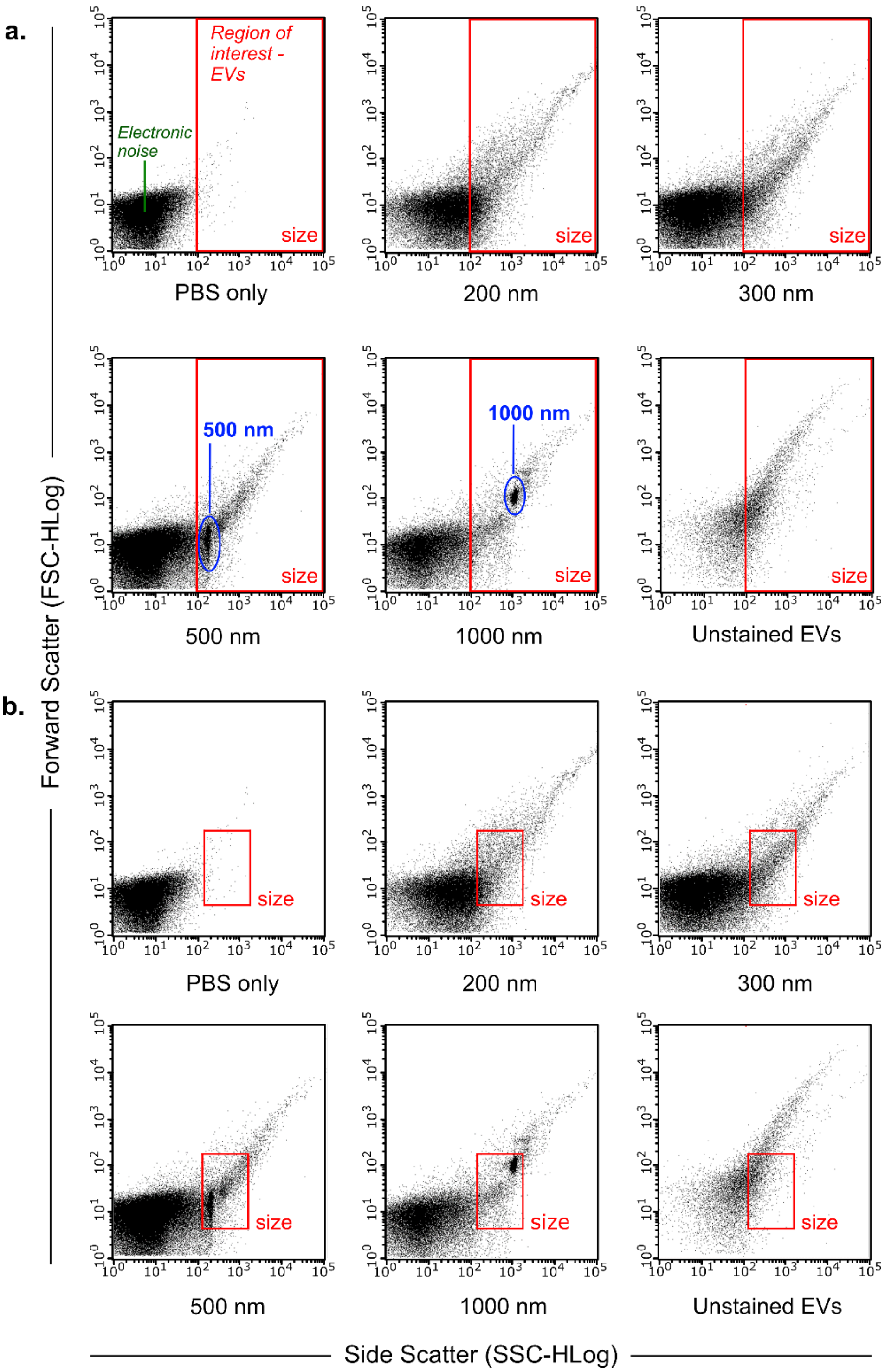

Figure S1. Flow cytometry gating strategy for the identification of intact large EVs (mainly microvesicles, MVs). **(a)** Various size of beads (200, 300, 500 and 1000 nm, NanoSpheres) diluted 50,000-fold with PBS (same as that used for dilution of EV) were used for size determination of EVs that can be detected by scattering as well as for standardization of the flow cytometry tests. A clear cluster of 500 and 1000 nm beads (as highlighted in blue ellipse) could be easily seen above the background electronic noise shown in the lower left corner in each plot, in terms of size (forward scatter) and granularity (side scatter). **(b)** Re-sizing to include 500-1000 nm region only. These results showed that the Guava flow cytometer can detect EVs with size ≥ 400 nm, which was consistent with the actual size distribution of EVs upon LPS stimulation in Figure 2.

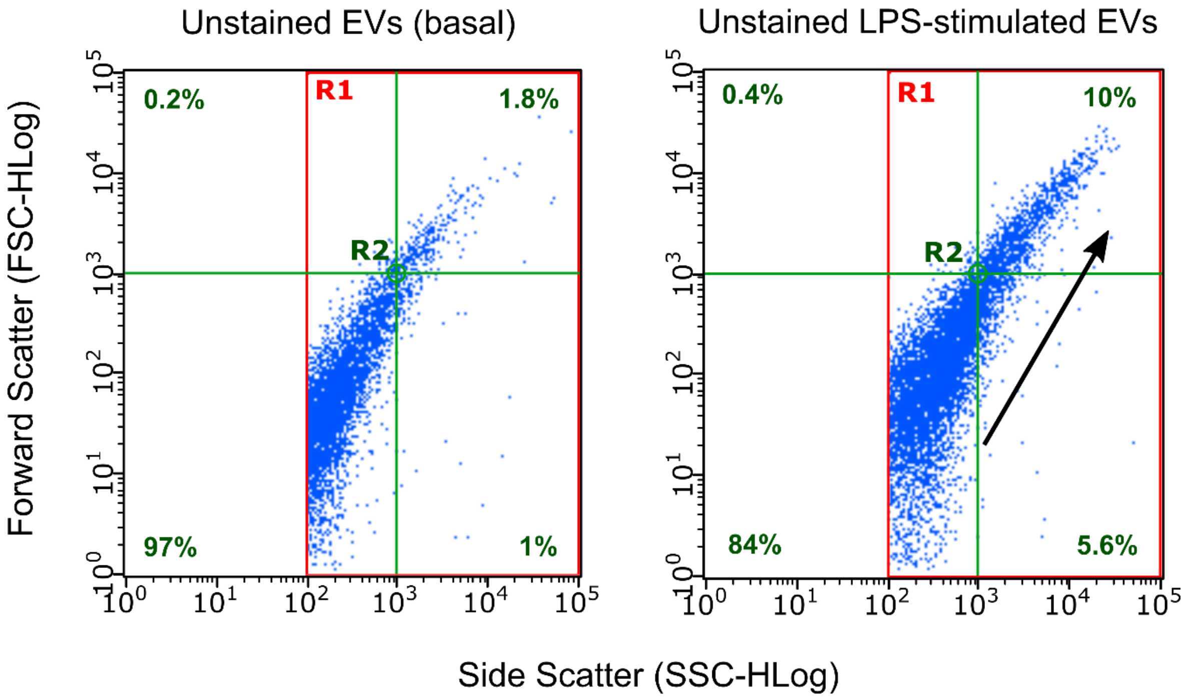

(Gate R1) *The region of interest - EVs*

(Gate R2) *The percentage of EV population in each quadrant within the region of interest (R1)*

Figure S2. Flow cytometry forward vs. side scatter intensity plots of unstimulated (basal) and LPS-stimulated EVs. EV population was gated based on FSC and SSC using a rectangle scale (R1, red outlined region, right panels) to gate out background “electronic noise”. The arrow indicates increased size and more numerous monocytic EVs.

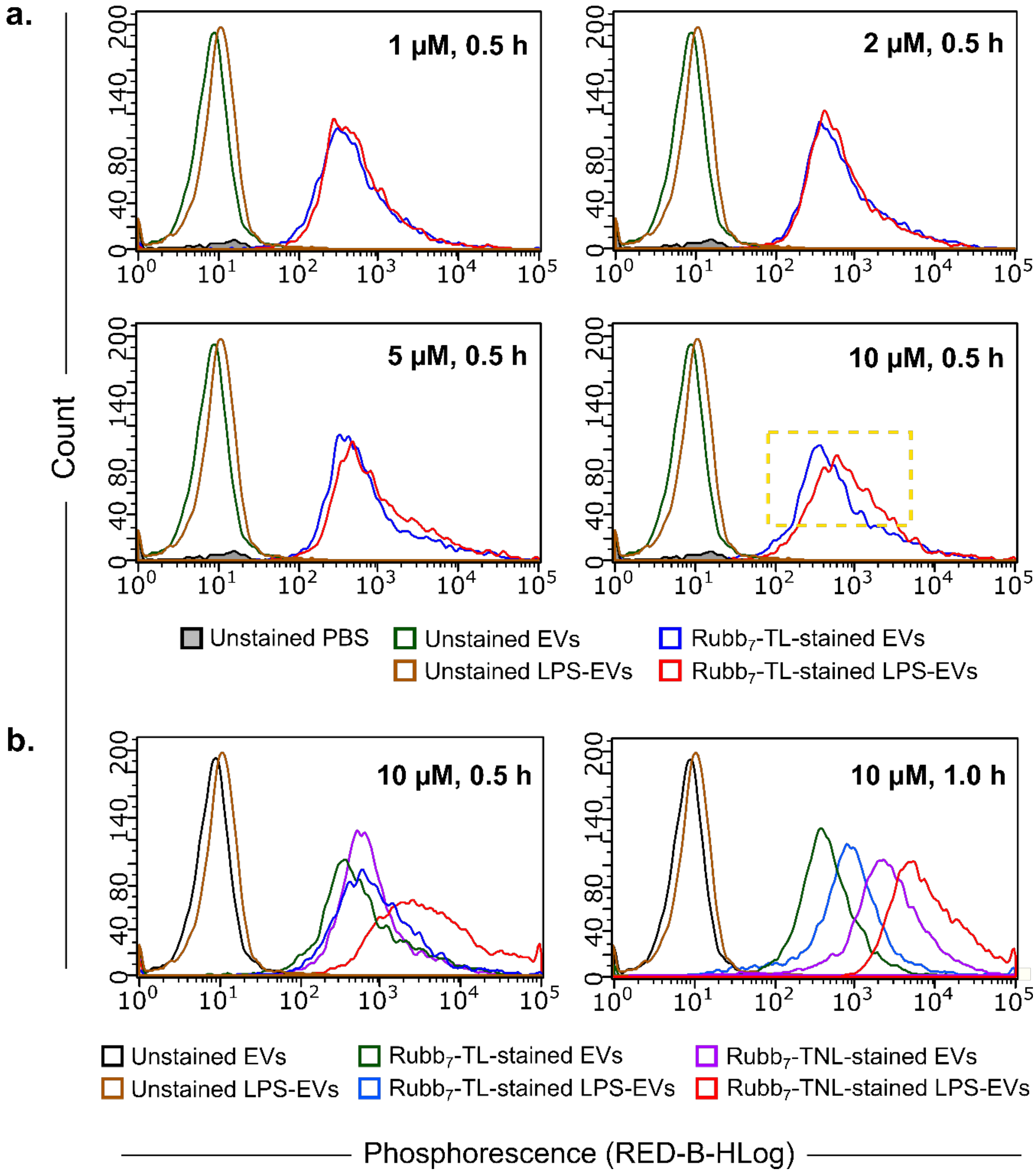

Figure S3. Optimization of staining conditions for monocytic EVs released from unstimulated and LPS-stimulated THP-1 cells. **(a)** Both unstimulated and LPS-stimulated EVs were stained by **Rubb$_7$-TL** (1, 2, 5 and 10 µM) in PBS for 0.5 h at 295 K, followed by removal of excess Ru-stain. Noticeable changes were observed with 10 µM as highlighted in yellow dashed rectangle that indicates the optimum concentration of dyes for staining EVs. **(b)** Both unstimulated and LPS-stimulated EVs were stained by 10 µM of each **Rubb$_7$-TNL** and **Rubb$_7$-TL** at different staining time (0.5 and 1 h).

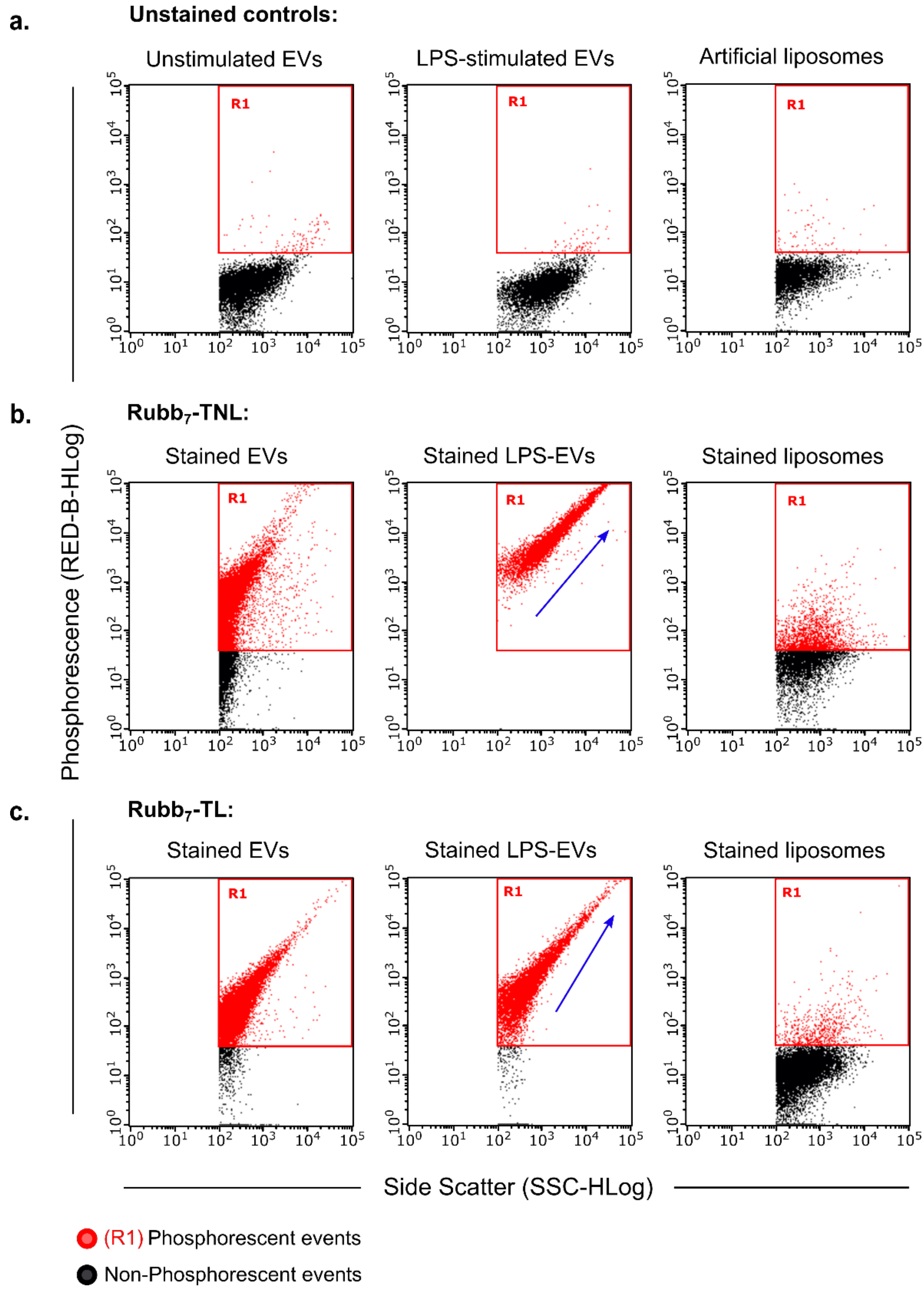

Figure S4. Selectivity of **Rubb$_7$-TNL** and **Rubb$_7$-TL** for the detection of intact monocytic EV-related nucleic acids. Comparison of phosphorescence vs. side scatter intensity plots for representative samples of: **(a)** unstained EVs from resting and LPS-stimulated THP-1 monocytes, and unstained artificial DOPC/cholesterol liposomes (suspensions in PBS): **(b)** **Rubb$_7$-TNL:** and **(c) Rubb$_7$-TL** in the presence of unstimulated EVs, LPS-stimulated EVs, and DOPC/cholesterol liposomes. A gate representing the size range of interest was set according to known diameters of standard beads and the instrument settings. Events within the gate or estimated size range of interest (R1, red outlined region, right upper channels), identified as **Rubb$_7$-TNL** or **Rubb$_7$-TL**-stained intact monocytic EVs. The arrows indicate increased size, more numerous monocytic EVs and higher phosphorescence intensity.
